# Association between kidney stones and risk of developing stroke: a meta-analysis

**DOI:** 10.1007/s10072-021-05113-5

**Published:** 2021-02-19

**Authors:** Min Yuan, Huang-Yan Zhou, Fan Hu, Shi-Ying Liu, Wei Rao, Ling-Feng Wu, Hong-Bing Nie, Wen-Feng Cao

**Affiliations:** 1 Department of Neurology, Jiangxi Provincial People’s Hospital Affiliated to Nanchang University, No. 152, Aiguo Road, Nanchang, 330006 Jiangxi China; 2 Department of Blood Transfusion, Jiangxi Cancer Hospital, Nanchang, 330029 Jiangxi China

**Keywords:** Kidney stones, Stroke, Cerebrovascular disease, Meta-analysis

## Abstract

**Background:**

Many studies have described the relationship between kidney stones and stroke, but the results are controversial, so we conducted this meta-analysis to estimate the relationship between kidney stones and the risk of developing stroke.

**Methods:**

Studies were marked with a comprehensive search of PubMed, EMBASE, Google, and ISI Web of Science databases through 25 March 2020. Hazard ratios (HRs) and 95% confidence intervals (CIs) were extracted, and a random-effects model or fix-effects model was used to compute the pooled combined risk estimate. Heterogeneity was reported as *I*^2^. We performed subgroup and sensitivity analysis to assess potential sources of heterogeneity.

**Results:**

Eight studies of seven articles involving 3,526,808 participants were included in the meta-analysis. Overall, kidney stones were associated with a moderate risk of stroke incidence (HR, 1.24; 95% CI, 1.11–1.40; *I*^2^=79.6%; *p*=0.000). We conducted a sensitivity analysis by removing the studies that had a high risk of bias. Heterogeneity subsequently decreased significantly, while an increased risk of stroke in patient with kidney stones was again demonstrated (HR, 1.16; 95% CI, 1.11–1.23; *I*^2^=28.7%; *p*=0.000). Stratifying analysis showed that the results were more pronounced for ischemic stroke (HR, 1.14; 95% CI, 1.08–1.22; *I*^2^=15.6%; *p*=0.00) and the follow-up duration ≥10 years (HR, 1.18; 95% CI, 1.10–1.27; *I*^2^=31.6%; *p*=0.003).

**Conclusions:**

Our meta-analysis suggests that patients with kidney stones may have a modestly increased risk of developing stroke, especially in ischemic stroke. More large-scaled and clinical trials should be done to identify the relative impact of kidney stones on stroke outcomes in the future.

**Supplementary Information:**

The online version contains supplementary material available at 10.1007/s10072-021-05113-5.

## Introduction

Stroke is a common cerebrovascular disease known for its high incidence, high mortality, and increased disability rate. Depending on statistics, it is the second-largest cause of death and disability in the world [1–3]. On average, someone died of a stroke every 4 min [4]. Studies showed that about 780,000 Americans experience a new or recurrent stroke every year [5]. In 2005, accounted for 5.7 million deaths, 16 million first-time stroke events worldwide, and it is speculated that these numbers may reach 7.8 million and 23 million by 2030 [5], respectively, according to estimates by the WHO, which creates a significant public health burden on the society. Therefore, identifying primary prevention for stroke risk and any possible means to prevent a stroke should be a critical public health priority, especially among young adults.

Kidney stones are a relatively prevalent disease in our daily life, especially in Western civilizations [6, 7]. Many studies have assessed the association of kidney stones with stroke risk [8–13]. However, the role of kidney stones in stroke is still controversial. A previous meta-analysis showed that kidney stones are associated with increased cardiovascular risk, but only three stroke studies were included [14]. Simultaneously, they did not analyze the influence of study design, stroke type, geographic area, follow-up time, and study quality. The results are also quite heterogeneous. Besides, Peng et al. [15] conducted a meta-analysis only in 4 studies, and the description of the subgroup analysis is inconsistent with that of Fig. 3. What is more, the data of meta-analysis was just limited to May 2016. Furthermore, we found several articles that they did not include [9, 13, 16]. Recent studies involving the relationship between kidney stones and risk of stroke incidence were published from then on [12]. To obtain a more comprehensive estimate of kidney stones’ putative influence on stroke, we conducted a meta-analysis of all related studies to determine the association between kidney stones and stroke risk.

## Materials and methods

### Literature search

Our meta-analysis was conducted and reported according to the recommendations of Preferred Reporting Items for Systematic Reviews and Meta-Analyses (PRISMA) Statement [17]. We searched PubMed, EMBASE, Google, and ISI Web of Science databases for the related published articles through 25 March 2020. We used the following keywords: “kidney stones,” “renal stones,” “renal calculus,” “kidney calculi,” “nephrolith,” “nephrolithiasis,” “stroke,” “cerebrovascular disorders,” “cerebrovascular accidents,” “cerebrovascular disease,” “cerebral infarct,” “ischemic stroke,” and “intracranial hemorrhage.” There were no any language restrictions.

### Study selection

Studies were included for our meta-analysis if they fulfilled the following criteria: (1) the study had a cohort design, case control, or cross-sectional design; (2) assignment of kidney stones as the baseline exposure, stroke was the outcome measure; and (3) the reported quantitative estimates of the multivariate-adjusted hazard ratios (HRs), odds ratio (OR) or relative risk (RR), and 95% confidence intervals (CIs) for stroke associated with kidney stones. The original effect size value (OR, RR) was used 1:1 as input for HR in the sense of best estimation.

### Data abstraction and quality assessment

All data were independently abstracted in duplicate using a standard data collection form. When necessary, the original authors were contacted for supplementary information. Discrepancies were settled by consensus. The following data were extracted from each study: first author’s last name, year of publication, study design, source of population, the country where the analysis was performed, size of the study, age range or average age, follow-up time, assessment of kidney stones and stroke, and study quality. The Newcastle–Ottawa Scale (NOS) was utilized to assess the quality of the studies [18]. The full score was 9 stars. If the score of studies met ≥7 awarded stars, it means high-quality studies.

### Statistical analysis

Stata 12 (Stata Corporation, College Station, TX) was utilized to perform the meta-analysis. *P* values were 2-sided, and *p* < 0.05 was considered statistically significant. For each study, the HR was used for the measurement data and presented with 95% confidence intervals. The chi-square test’s degree of heterogeneity among the results was estimated (the *p* value < 0.1 was considered significant). Whenever significant heterogeneity (*p* value < 0.10 or *I*^2^ score > 50%) was achieved, the random-effects model was used. If no significant heterogeneity across studies was found, a fixed-effect model was selected to pool the data [19]. The pStatemenias was identified with the symmetry of the funnel plot, Egger’s test, and Begg’s test. Sensitivity analyses and subgroups were performed where appropriate.

## Results

### Literature search

**Fig. 1:**
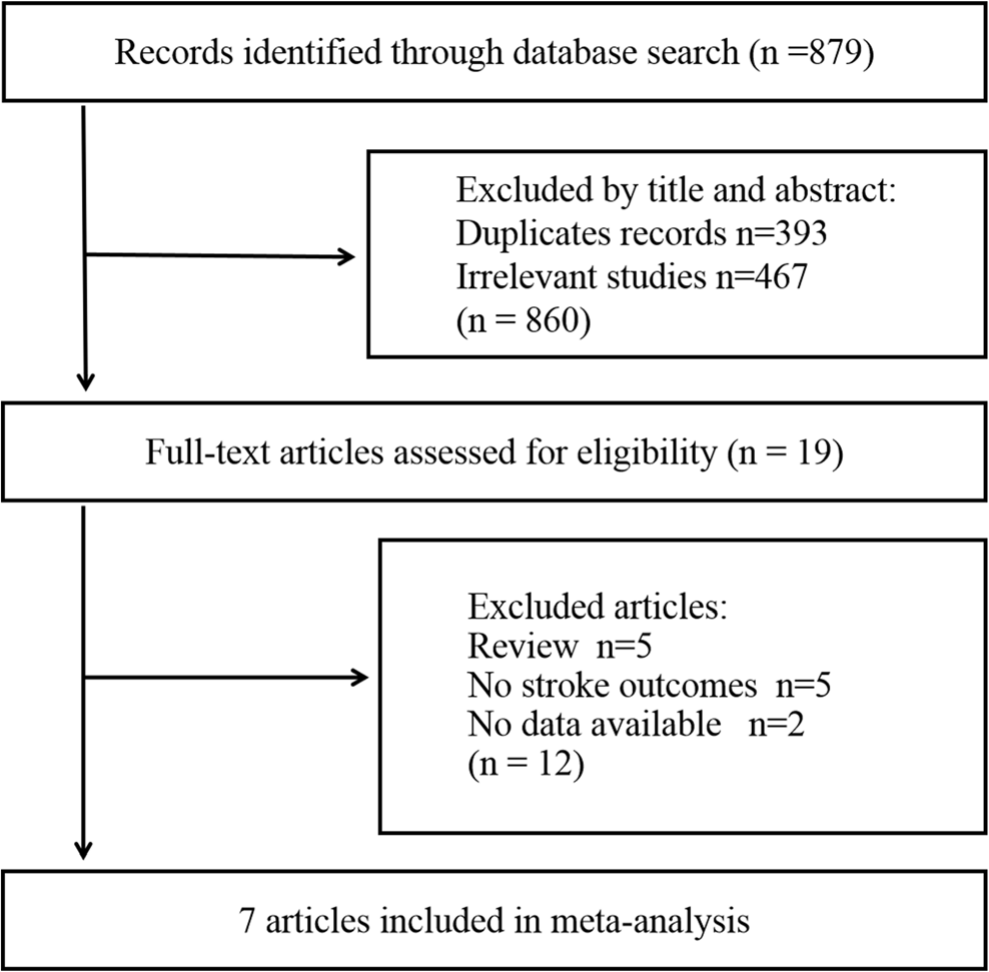
Flow chart of study selection

A flow chart displaying the results of the literature search and selection is presented in Fig. 1. We initially identified 879 potential publications through the systematic retrieval of PubMed, EMBASE, Google, and ISI Web of Science databases. After excluding duplicate records and studies or after scanning the title and abstract that did not fulfill our inclusion criteria, 19 studies remained. We evaluated the full texts of these 19 publications and excluded 12 studies for the following reasons: reviews (*n*=5) [18–24], no stroke outcomes (*n*=5) [25–29], and no data available (*n*=2) [30, 31]. Seven articles in eight studies finally met the inclusion criteria and were included in the meta-analysis [8–13, 16].

### Study characteristics

The characteristics of the studies and their participants are presented in Table 1. Seven articles from eight studies involving 3,526,808 participants were included in the meta-analysis. Of these seven articles [8–13, 16], there are two research results in each of two articles [10, 12]. Alexander et al. [10] elaborated on cardiovascular events in the primary and laboratory cohorts. Kim et al. [12] described the cohort studies of ischemic stroke and hemorrhagic stroke, and, respectively, all have available data. However, the “laboratory cohort” is not an independent group of participants; it is a mere subset from the “primary cohort,” so the data were not included in the analysis. Three studies were conducted in Asia [8, 9, 12]; three studies were conducted in Europe [11, 13, 16], and one in a North American country (Canada) [10]. Six articles are cohort studies [8–13], and one is a cross-sectional study [16]. Only three of the seven articles identified ischemic stroke [8, 12, 13], and one pointed to hemorrhagic stroke [12], and data were available. All studies included both men and women, but no specific operational data. The duration of follow-up varied from 1.1 to 12 years, with an average of 7.6 years. The assessment of kidney stones varied across studies. In most studies, kidney stones were measured using International Classification of Diseases-9-CM (ICD-9-CM) [12] and ICD-10 [12]; three studies were measured using a self-reported questionnaire as well as clinical examinations [11, 13, 16]. In most studies, stroke was evaluated by medical records based on ICD-9 or ICD-10. Only one study was measured using self-reported questionnaire [16]. All studies provided risk estimates adjusted for related covariance; the quality score of studies ranged from 6 to 9, and the average score was 7.7. Among them, there were five studies [8–10, 12, 13] with scores ≥ 7.

**Table 1:** Characteristics of studies included in this meta-analysis

Author, publication (year)	Study design	Source of population	Country/population	Participants	Age range or average age	Follow-up duration (year)	Assessment of kidney stone	Stroke ascertainment	Adjustment for covariates	Study quality
Chung et al (2012)	Prospective cohort study	Taiwan Longitudinal Health Insurance Database 2000	Taiwan/Asian	134,097	44.3	5	ICD-9-CM	ICD-9-CM	Age, sex, monthly income, urbanization level, BMI, HTN, diabetes, gout, race, CHD, hyperlipidemia, the year of index date, heart failure, year of index ambulatory care visit, atrial fibrillation	7
Alexander et al (2014)	Prospective cohort study	Alberta Kidney Disease Network database	Canada/North American	3,195,452	40.6	11	Physician claims, hospitalization, ambulatory care utilization data, and ICD-9-CM	ICD-9-CM, ICD-10-CA I21, and I22 hospitalization claims	Age, sex, diabetes, social assistance, race, region of residence, distance to nearest nephrologist, hyperlipidemia, smoking, alcohol, chronic kidney disease, BMI, HTN	9
Wirth et al (2014)	Prospective cohort study	The European Prospective Investigation into Cancer and Nutrition-Potsdam study	German/European	24,490	35–65	8.1	Via questionnaires	ICD-10	Age and sex, education, physical activity, smoking, alcohol intake, BMI, waist circumference, prevalent hypertension and prevalent diabetes	8
Hsu et al (2016)	Prospective cohort study	Longitudinal Health Insurance Database from 1995 to 2010	Taiwan/Asian	25,372	46.4	10	ICD-9-CM	ICD-9-CM	Age, gender, medical care utilization as calculated by ambulatory visits during the past 1 year, income, and level of urbanization	9
Kim et al (2019)	Cohort study	Korean Health Insurance Review and Assessment Service-National Sample Cohort	Korean/Asian	113,180	≥ 20	12	ICD-10	ICD-10 codes	Age, sex, income, region of residence, hypertension, diabetes, hyperlipidemia, ischemic heart disease, and depression histories	9
Domingos et al (2011)	Cross-sectional study	The Portuguese National Health Survey	Portugal/European	23,349	≥15	1.1	Via questionnaires	Via questionnaires	Age and BMI	6

*ICD* International Classification of Diseases, *BMI* body mass index, *HTN* hypertension, *CHD* coronary heart disease

### Kidney stones and stroke incidence

**Fig. 2:**
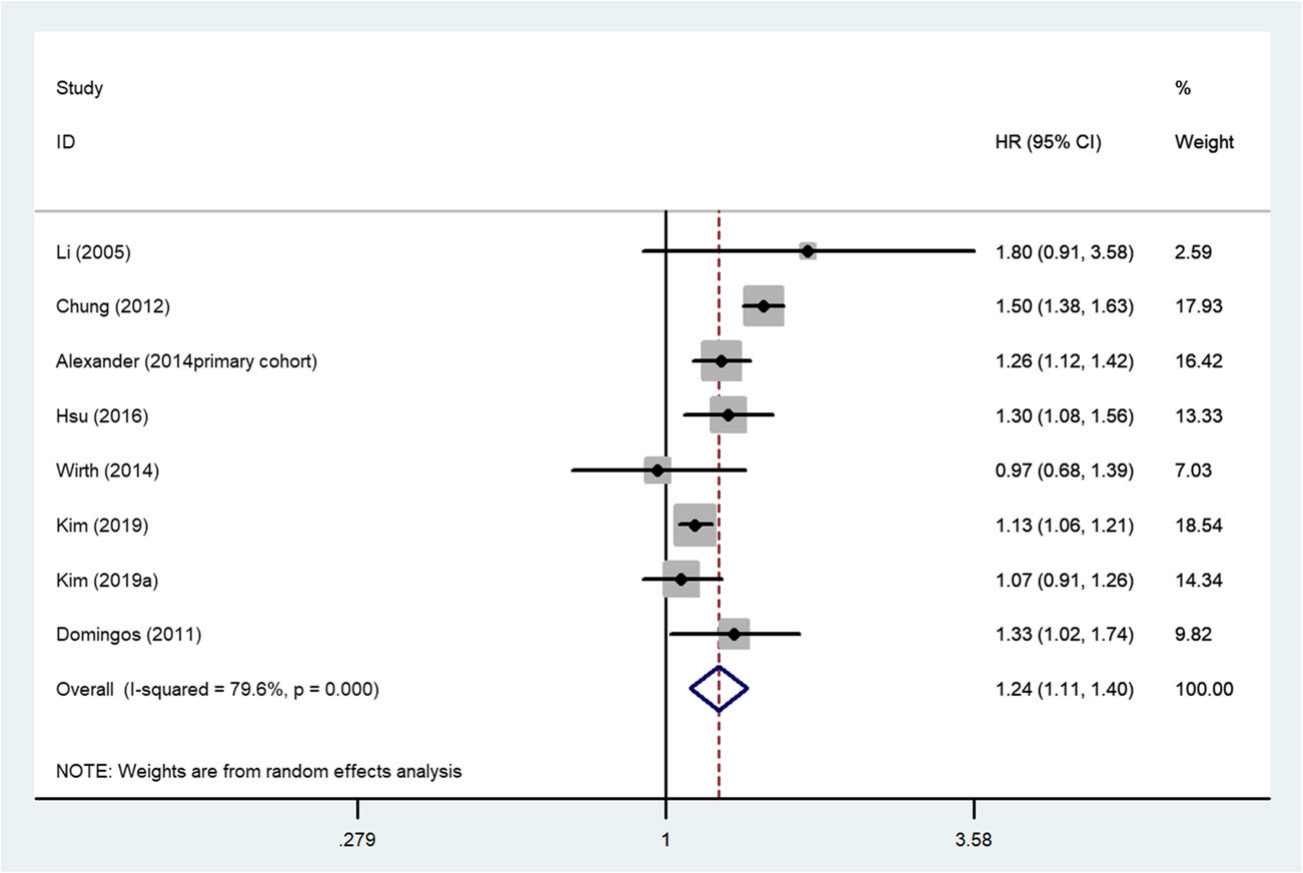
Random-effects analysis of fully adjusted studies for the association between kidney stones and stroke risk

Eight studies of seven articles [8–13, 16] elaborated the relationship between kidney stones and stroke. We proceeded to a meta-analysis of all the data. From the results of the analysis, there was statistically significant heterogeneity in our study (*I*^2^=79.6%), so we used the random-effects model. Multivariable-adjusted HR of stroke incidence concerning kidney stone from individual studies is presented in Fig. 2. Participants with kidney stones experienced a moderately increased risk for stroke development based on eight studies compared with non-kidney stone (HR, 1.24; 95% CI, 1.11–1.40; *p* = 0.000). When we excluded the cross-sectional study [16] and analyzed all cohort studies, the combined HR was 1.24; 95% CI, 1.09–1.40; *p* = 0.001. The result was statistically significant, confirming the increased risk of stroke in patients with kidney stones again.

### Stratifying analysis

There was statistically significant heterogeneity in the overall analysis, and the source of population, duration of follow-up, study design, and methodological quality varied across studies. Therefore, we also conducted subgroup analyses further to elucidate the effect of kidney stones on stroke risk. Table 2 showed pooled HR for stroke stratified by study design, geographical area, stroke type, duration of follow-up, and study quality. The HR for Asian and North American studies indicated that kidney stones were associated with an increased risk of stroke (Asian: 1.24 (95% CI, 1.04–1.48; *p* = 0.014; North American: 1.26 (95% CI, 1.12–1.42; *p* = 0.000), while Europe studies were not associated with a risk of stroke (HR, 1.24 (95% CI, 0.93–1.64; *p* = 0.141). The pooled estimate of multivariate HRs based on three studies [8, 12, 13] was 1.14 (95% CI, 1.08–1.22; *p* = 0.000) among ischemic stroke and 1.07 among hemorrhagic stroke (95% CI, 0.91–1.26; *p* = 0.415) based on one study [12]. These results suggest that kidney stones are associated with the risk of ischemic stroke. Besides, increases in stroke events were also found in the subgroup meta-analysis of study quality (≥7 or <7) and duration of follow-up (≥10 years), which indicated that kidney stones are closely associated with an increased risk of stroke regardless of study quality.

**Table 2:** Stratified analyses of kidney stones and stroke incidence

					Heterogeneity test			
Subgroup	Categories	No. of cohorts	Adjusted HR	95% Cl	*X*^2^	*p* **value**	*I*^2^, %	*p* value of pooled effect
Study design	Cohort study	7	1.24	1.09–1.40	34.05	0.000	82.4	0.001
	Cross-sectional study	1	1.33	1.02–1.74	0.00	.	.	0.036
Geographical area	Asian	4	1.24	1.04–1.48	31.03	0.000	90.3	0.014
	Europe	3	1.24	0.93–1.64	3.21	0.201	37.7	0.141
	North America	1	1.26	1.12–1.42	0.00	.	.	0.000
Stroke type	Ischemic stroke	3	1.14	1.08–1.22	2.37	0.306	15.6	0.000
	Hemorrhagic stroke	1	1.07	0.91–1.26	0.00	.	.	0.415
Quality score	<7	2	1.38	1.08–1.78	0.65	0.420	0.0	0.010
	≥7	6	1.22	1.07–1.39	32.93	0.000	84.8	0.003
Follow-up duration (year)	<10	3	1.34	0.97–1.86	5.76	0.056	65.3	0.079
	≥10	5	1.18	1.10–1.27	5.85	0.211	31.6	0.003

### Sensitivity analysis and publication bias

**Fig. 3:**
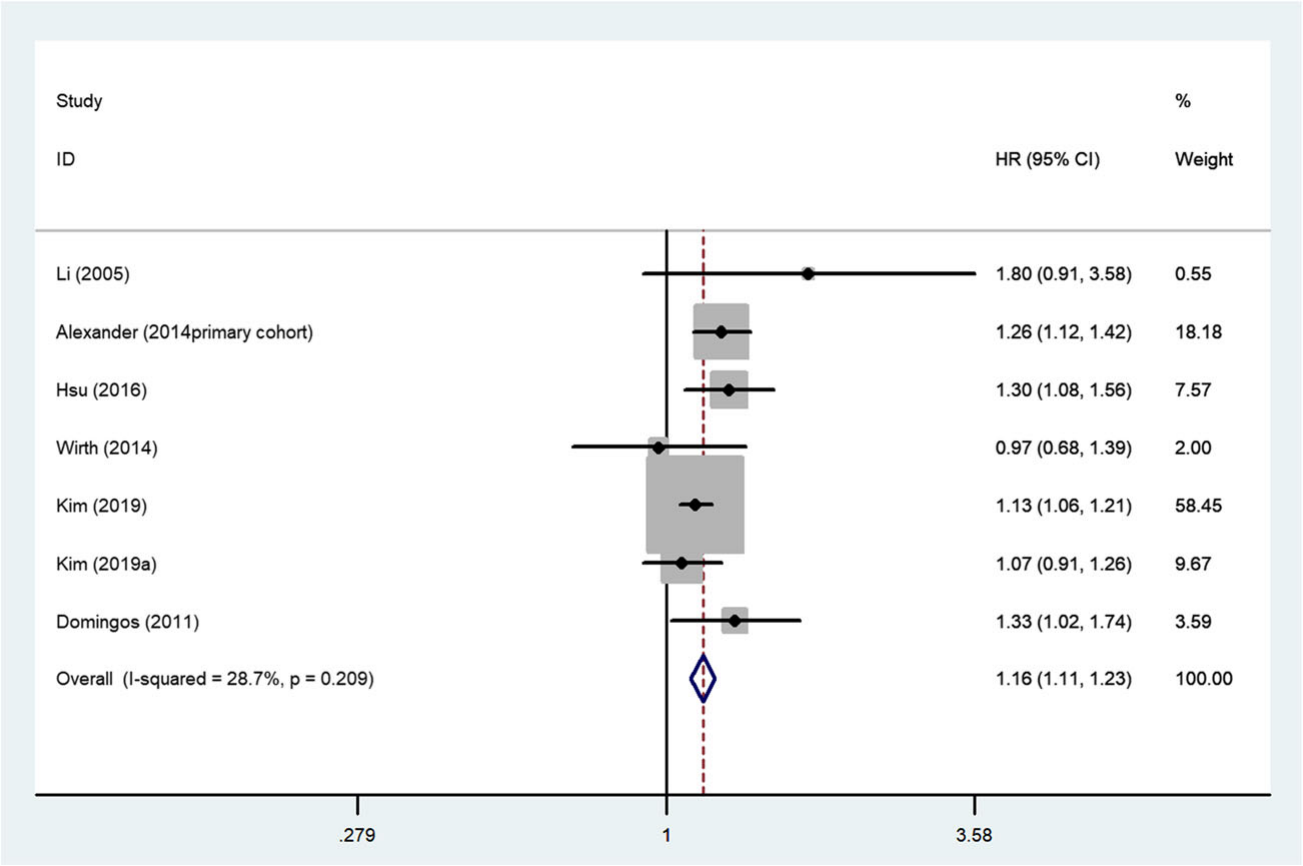
Fixed-effects analysis of fully adjusted results for the association between kidney stones and stroke risk after sensitivity analysis

**Fig. 4:**
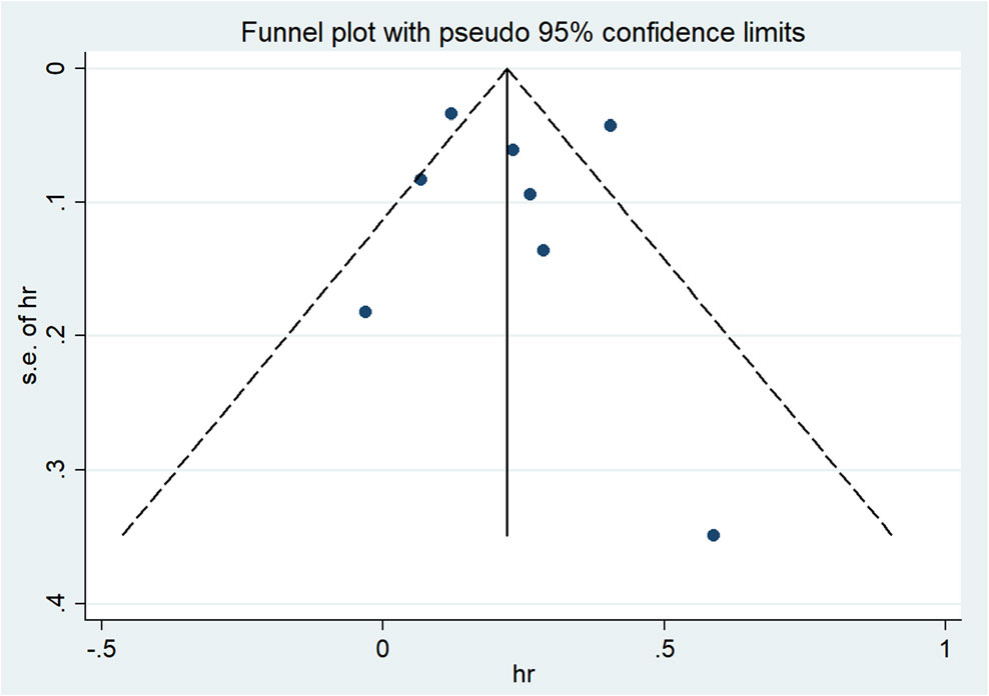
Funnel plots of kidney stones and the risk of stroke incidence

We tested the robustness of our results in a sensitivity analysis by omitting one study at one time. The results showed that the study of Chung et al. [9] substantially affected the pooled HRs. When we deleted the study and calculated the pooled HRs with the fixed-effect model for the remaining of the studies, significant increases in stroke events were also found: HR, 1.16 (95% CI, 1.11–1.23; *p*=0.000), the heterogeneity was significantly decreased (*I*^2^=28.7%, *p*=0.209) (Fig. 3). There was no evidence of publication bias by inspection of the funnel plot (Fig. 4). Further study showed no evidence of substantial publication bias was observed among studies for stroke risk from the Begg’s test (*p*=0.322) and Egger’s test (*p*=0.929).

## Discussion

Our meta-analysis from eight studies of seven articles confirmed a positive association between kidney stones and the stroke risk after adjusting established cardiovascular risk factors, with an overall 1.24-fold increased risk compared with those without a history of kidney stones.

Over the past decades, despite extensive studies investigating the kidney stone’s role on either cardiovascular disease or stroke, it remains unclear whether the association between kidney stones and risk of stroke incidence is causal. Some of the studies suggested that kidney stones were associated with an increased risk of stroke, and the others failed to find the association. Many studies showed that kidney stones are associated with coronary heart disease (CHD) [10, 32], hypertension [33], diabetes [34], atherosclerosis [28], and metabolic syndrome [35], which are the risk factor for CHD and stroke; thus, patients with nephrolithiasis might have a high stroke risk. Through a 10-year follow-up of people in Taiwan, Hsu et al. [8] found a positive correlation between the increased risk of kidney stones and stroke, which is consistent with previous epidemiological evidence [9, 10, 16, 31] and the results of our current study. Lin et al. [30] analyzed data from a large number of patients with nephrolithiasis. They matched controls from a national insurance claim dataset of 22 million enrollees in Taiwan with 13 years of follow-up. They found that nephrolithiasis is associated with an increased risk of ischemic stroke, particularly for women and the younger population, based on the population-based study. This is consistent with our study’s subgroup analysis showing that kidney stones are associated with the risk of ischemic stroke, but there is no significant correlation for hemorrhagic stroke. However, because this article does not provide useful data after adjusting the established risk factors, we omit this article in the study. However, Li et al. [11] and Wirth et al. [13] reported that patients with renal calculus did not associate with stroke incidence. Moreover, the laboratory cohort and primary cohort studies conducted by Alexander et al. [10], including 3,195,452 participants, showed two different results. Meanwhile, Kim et al. [12] found differences in ischemic stroke incidence and hemorrhagic stroke in patients with kidney stones in a cohort of 113,180 participants in 2019. Therefore, we carried out a meta-analysis of all related studies to quantitatively identify whether kidney stones are associated with the risk of stroke and eliminate these disputes as much as possible, guiding the primary prevention of stroke patients in the clinic.

For the moment, the mechanisms underlying the increase of consequent stroke events in kidney stones patients are not well understood. Previous studies showed that kidney stones are associated with hypertension, smoking, obesity, diabetes, and hyperlipidemia, which are all known risk factors for stroke [33, 34, 36], and kidney stone formers had increased evidence of subclinical atherosclerosis with the pathogenesis of stroke event [28]. Other studies have shown that obesity and insulin resistance can lead to poor ammoniation, so diabetes can increase the risk of uric acid kidney stones by inducing low urine pH [37]. Furthermore, lots of kidney stones are composed of calcium. Hypercalciuria should be the most significant risk factor for the development of kidney stones [26, 38]. In contrast, patients with increased calcium precipitation in parts of the body, such as the intracranial region and coronary vessels, may link the underlying pathophysiology of the formation of calcium precipitations in the renal tubule, which would, in turn, cause the clinical sequelae of stroke [39].

However, heterogeneity between studies was found in kidney stones and risk of stroke incidence. Therefore, we chose the random-effects model to analyze the results; stratifying analysis and sensitivity analysis were also used to reduce heterogeneity and find the source of heterogeneity. Within the stratifying analysis, we examined study design, geographical area, stroke type, length of follow-up, and study quality as possible sources of heterogeneity, and some studies showed that these did not show any significant heterogeneity between studies. Although the stratifying analysis could not explain the heterogeneity level in interpreting the results, several studies’ differences are worth discussing. When we conducted a sensitivity analysis by omitting one study at one time, the results showed that the study of Chung et al. [9] substantially affected the pooled HRs. When we deleted the study and calculated the pooled HRs for the remaining of the studies, the heterogeneity was significantly decreased (HR, 1.16; 95% CI, 1.11–1.23; *I*^2^=28.7%; *p*=0.000), we found that the study was a prospective cohort study, which was conducted by the source from the Taiwan Longitudinal Health Insurance Database 2000. The diagnosis of kidney stone and stroke in this article depends on the doctors’ and hospitals’ data, rather than based on standardized criteria, and residual confounding factors and surveillance biases also existed. As we all know, according to the GRADE system, five factors may lead to a decrease in the quality of evidence, including the risk of bias, inconsistent results, indirectness of evidence, inaccuracy of the results, and publication bias. In this article, we believe that the inconsistency in rating the quality of evidence is the main reason for the current results and heterogeneity. Of course, differences in environmental factors, countries, methodological factors in study design, and how the studies were conducted should attribute to the observed heterogeneity. Therefore, the existence of heterogeneity requires a proper and careful understanding of the current meta-analysis findings.

This study has several important strengths compared with previous meta-analyses. To our knowledge, this article provides a more systematic explanation of the relationship between kidney stones and stroke risk. It also conducts stratifying analysis on some known influencing factors. At the same time, sensitivity analysis is used to screen the research results’ heterogeneity. Furthermore, the Newcastle-Ottawa Scale was used to assess individual studies’ quality. Most of them were of high quality. Besides, we use the funnel chart to directly show that there is no publication bias in the research results, which are further confirmed by Begg’s and Egger’s tests. Therefore, the results should be reliable.

There were also many limitations to our meta-analysis. Firstly, a limited number of studies were included, although we strove to find all related studies. Only eight studies of seven articles were included in our meta-analysis. Secondly, although we considered a multitude of risk factors in the multivariable analysis, the possibility of residual confounding or confounding by unmeasured factors, which cannot be ruled out in any observational study, must be acknowledged. Thirdly, although we used the random-effects model and stratifying analysis, the results still have some heterogeneity. Also, the number of studies included in the subgroup was small, lacking sufficient reliability to confirm a relationship in a definitive manner. The number of available studies of different stroke outcomes and differences in study design, country, and assessment method included in this meta-analysis was moderate. Therefore, the results could be influenced by factors like methodological differences, regional differences, etc.

## Conclusion

Our meta-analysis found a moderate association between kidney stones and the risk of stroke incidence after adjustment of established cardiovascular risk factors, especially in ischemic stroke. To efficiently assess the association and causality of kidney stones on stroke, more large-scaled and clinical trials should be done to identify the relative impact of kidney stones on stroke outcomes and explore whether effective treatment of kidney stones may prevent or improve the course of stroke.

## Supplementary information

ESM 1 (DOC 60 kb)

## Data Availability

All primary studies surveyed are published. To the best of our knowledge, no unpublished studies were available.
